# Magnetic resonance imaging versus musculoskeletal ultrasonography in detecting inflammatory arthropathy in systemic sclerosis patients with hand arthralgia

**DOI:** 10.1007/s00296-013-2665-8

**Published:** 2013-01-25

**Authors:** Rasha A. Abdel-Magied, A. Lotfi, Ehab A. AbdelGawad

**Affiliations:** 1 Department of Rheumatology and Rehabilitation, Minia University, Minia, Egypt; 2 Department of Diagnostic Radiology, Minia University, Minia, Egypt

**Keywords:** Scleroderma, Hand arthralgia, MSUS, MRI

## Abstract

The aim of the study was the detection of inflammatory arthropathy in patients with systemic sclerosis (SSc) with arthralgia using musculoskeletal ultrasonography (MSUS) and magnetic resonance imaging (MRI) and to compare between MRI versus MSUS detecting musculoskeletal abnormalities and find out its relation with other clinical and laboratory parameters. Sixteen SSc patients with hand arthralgia had a baseline MSUS for their hands. Six months later, patients had a second MSUS and MRI with gadolinium of their most symptomatic hand. Of the 16 patients examined by MSUS, it was found that on baseline and second examination, tenosynovitis was seen in 8 (50 %) and 7 (43.7 %) patients and synovitis was seen in 4 (25 %) and 5 (31 %) patients, respectively, indicating persistence synovial inflammation, and erosion was seen in only 1 (6.3 %) patient on baseline and second examination. Regarding MRI, 81.3 % (13) patients had tenosynovitis, 87.5 % (14) patients had synovitis, and 62.5 % (10) patients had erosions. Applying the RAMRIS system (a semiquantitative MRI scoring system used in RA), the mean values for synovitis, bone marrow edema, and erosions fell within the range seen in RA. Systemic sclerosis patients with arthralgia that have no obvious clinical inflammatory arthritis were found to have persistent inflammatory erosive arthropathy in their hands and wrists using MSUS and MRI. While both MRI and MSUS are useful in characterizing synovial inflammation in SSc, MRI is clearly more sensitive than MSUS in this setting. Further studies on larger number of SSc patients with arthralgia and a control group consisting of SSc patients without arthralgia to better establish the clinical and radiological findings in SSc.

## Introduction

Systemic sclerosis (SSc; scleroderma) is a chronic disorder of connective tissue characterized by inflammation, fibrosis, and degenerative changes in the blood vessels, skin, synovium, skeletal muscle, and multiple internal organs. The clinical features, organ system involvement, natural history, and survival among patients with SSc are highly variable and largely depend on SSc clinical subtype and SSc-associated serum autoantibodies [1]. The musculoskeletal findings in progressive systemic sclerosis have been the subject of continued controversy, partly because most reports describe mixed populations of SSc, mixed connective tissue disease (MCTD), overlap syndromes of several connective tissue diseases and calcinosis, Raynaud’s phenomenon, esophageal dysmotility, sclerodactyly, and telangiectasia (CREST) patients [2].

Generalized arthralgia and morning stiffness are typical symptoms of systemic sclerosis and may be confused with early RA [3]. Although arthralgia is common in SSc, its cause is poorly understood. It is usually attributed to mechanical factors resulting from fibrosis, with tendon friction [4]. Clinically appreciable joint inflammation is uncommon, although erosive arthropathy has been demonstrated to occur in some series in as many as 29 % of patients. Inflammatory and fibrinous involvement of tendon sheaths may mimic arthritis [3].

Erosive changes have been reported on X-rays in some SSc patients [5] and have been attributed to overlap with mixed CTD [6] or RA.

Musculoskeletal ultrasonography (MSUS) is used in the assessment of patients with inflammatory arthritis. This includes the detection of bone erosions, synovitis, and tendon disease. MSUS has a number of distinct advantages over magnetic resonance imaging (MRI), including its ability to scan multiple joints in a brief period of time and patient tolerability. MSUS, however, is often perceived as an imperfect and operator-dependent tool [7]; however, MRI is superior to MSUS in detection of inflammatory arthritis [4, 8, 9].

Musculoskeletal ultrasonography and MRI can identify and characterize subclinical synovial inflammation and joint damage with much greater precision than X-rays [8, 9].

## Patients and methods

Twenty patients meeting ACR classification criteria for SSc [10] without clinical features of overlap syndromes or MCTD were screened for arthralgia.

Patients with scleroderma/RA overlap or patients with MCTD were excluded from the study.

All the twenty patients underwent musculoskeletal examination and laboratory tests in the form of RF and anti-CCP antibodies. At the time of the start of the study, patients with swollen joints (clinical arthritis) and/or positive RF and/or positive anti-CCP serology were excluded to allow a reasonable assumption that our patients did not include patients with clinically or laboratory RA overlap.

Of these 20 patients who reported arthralgia, four patients were excluded from the study [2 patients had clinical arthritis (swollen joints) and positive anti-CCP antibodies, and the other 2 patients had positive RF]. After exclusion of those 4 patients, 16 patients were participated who were both RF and anti-CCP negative and did not have clinical arthritis. All patients participating in the study provided written informed consent.

All patients had detailed history (including age, sex, and disease duration) and a full clinical assessment of skin involvement and the musculoskeletal system. All patients had arthralgia, but none had any symptoms or signs of inflammatory arthritis (patients who had arthritis were excluded from the study). Laboratory evaluation including ESR, CRP, ANA, and anti-RNP was performed for all the patients.

Plain X-ray of both hands and wrists was done for all the studied patients.

Musculoskeletal ultrasonography evaluation was performed using Picus 4D GE Vivid-3 Expert machines, with 7.5–12 MHz phased-array transducer. Two rheumatologists experienced in MSUS sequentially performed scans of both wrists and hands assessing joints (radiocarpal, inter-carpal, MCP, PIP, and DIP) and tendons (all extensor and flexors of the fingers at the level of the wrists) using a multiplanar and dynamic scanning technique according to standard ultrasonographic scans proposed by the EULAR working group for MSUS in rheumatology [11].

All explored joints and tendons were evaluated for the presence of synovial inflammation and synovial hypertrophy on grayscale and synovitis/tenosynovitis on power Doppler ultrasonography (PDUS) signal according to OMERACT definitions criteria [7]. The presence of synovitis/tenosynovitis on grayscale and PDUS signal suggested synovial inflammation on MSUS, as per previously published guidelines [12].

All the 16 patients had a second MSUS of both hands after a 6-month interval performed by the same sonographists to look for persistence of synovial inflammation.

All the 16 patients had MRI scan with IV gadolinium contrast of their most symptomatic hand and wrist, within the same week of having the second MSUS. MRI was performed with a 1 T Magnet (Intera, Philips Medical Systems, Neberland B.V) with dedicated peripheral coils. IV gadolinium was used (Dotarem 0.5 mmol/ml). The following sequences were acquired: T1 weighted (TR 500, TE20, FOV 110 mm, Matrix 304, slice thickness 2.5 mm), fast spin-echo PD-weighted (TR 1800, TE37, FOV 110, Matrix 304, slice thickness 2.5 mm), and fat-suppressed images (TR 3500, TE 55, FOV 130, Matrix 272, slice thickness 3 mm).

MRI images were assessed by a musculoskeletal radiologist (blinded to the ultrasound findings) for the presence of synovitis, tenosynovitis, bone marrow edema, and erosions. Images were scored using the scoring system for synovitis, erosions, and bone marrow edema, used to score MRI scans in RA (RAMRIS) [13].

Statistical analysis was performed using SPSS for Windows version 17.0, two-tailed tests were used throughout, and statistical significance was set at <0.05 levels.

The following statistics were carried out: Descriptive statistics of the range, means, and standard deviation were calculated for interval and ordinary variables and frequencies and percentages for categorical variables, correlations (bivariate correlations procedure computes Pearson’s correlation coefficient with its significance levels), and percentage of agreement.

## Results

### Patients’ demographics and disease characteristics

Of the 16 patients involved in the study, there were 11 (68.8 %) with limited SSc and 5 (12.5 %) had diffuse SSc. There were 2 (12.5 %) males and 14 (87.5 %) females. Their mean age was 40.6 years (range 20–63 years), and their mean disease duration was 5.4 years (range 1–15 years). Among the 16 patients, CRP was positive in 11 (68.8 %) patients, ANA in 5 (93.7 %) patients, and anti-RNP in 2 (12.5 %) patients. All patients were RF and anti-CCP negative. The patient demographics and disease characteristics are presented in Table 1.

**Table 1:** Patients’ demographics and disease characteristics of the studied patients

	Range	Mean ± SD
Age (years)	20–63	40.6 ± 12.1
Disease duration (years)	1–15	5.4 ± 3.6
ESR (1st h)	16–46	30 ± 9.7

	Number	**%**
Sex		
Male	2	12.5
Female	14	87.5
Scleroderma type		
Limited	11	68.8
Diffuse	5	31.2
CRP		
Positive	11	68.8
Negative	5	31.2
ANA		
Positive	15	93.7
Negative	1	6.3
Anti-RNP		
Positive	2	12.5
Negative	14	87.5

Plain radiography of both hands and wrists did not show any evidence of erosions.

### MSUS findings

**Fig. 1:**
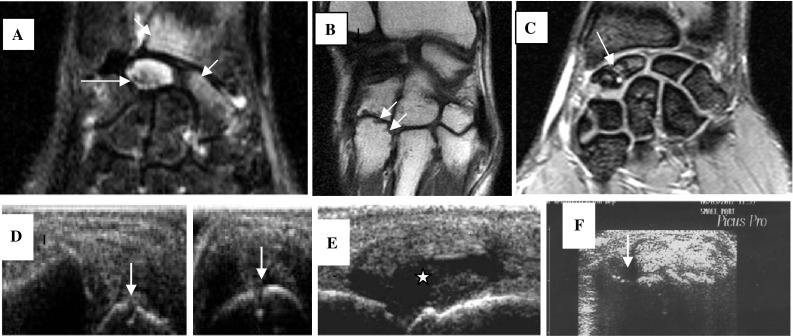
Imaging evidence of synovitis, tenosynovitis, and erosion. **a** Coronal STIR image shows an abnormal marrow signal involving the lunate bone (*long arrow*), also a hyperintense signal also seen partially affecting the scaphoid and the inner aspect of the radius denoting bone marrow edema (*short arrows*). **b** Coronal MRI image in a different patient shows erosive changes affecting the base of the 4th metacarpal bone (*arrows*). **c** Coronal T2 image shows erosive changes and marrow edema affecting the scaphoid bone (*arrow*). **d** MSUS image of bone erosion on longitudinal and transverse scan (*arrow*). **e** Longitudinal ultrasonographic view of MCP joint depicting grayscale synovial hypertrophy and synovitis (*asterisk*). **f** A transverse ultrasonographic view of the common extensor tendon at the level of the wrist showing grayscale tenosynovitis in the form of effusion (*arrow*)

At the baseline examination, tenosynovitis was present in 8 (50 %) out of 16 patients, synovitis was present in 4 (25 %) patients, and erosions were found in only 1 (6.3 %) patient. On the second MSUS examination after 6 months, tenosynovitis was present in 7 (43.8 %) out of 16 patients, synovitis was present in 5 (31.3 %) patients, and erosions were found in only 1 (6.3 %) patient (Fig. 1).

### MRI findings

Of the 16 patients examined by MRI on the same week with the second MSUS examination, tenosynovitis was present in 13 (81.3 %) out of 16 patients, synovitis was present in 14 (87.5 %) patients, bone marrow edema was present in 12 (75 %) patients, and erosions were found in 10 (62.5 %) patients (Fig. 1).

MSUS findings (tenosynovitis, synovitis, and erosion) and MRI findings (tenosynovitis, synovitis, erosion, and bone marrow edema) are presented in Table 2.

**Table 2:** MSUS and MRI findings

	MSUS at baseline	MSUS after 6 months	MRI
	Number (%)	Number (%)	Number (%)
Tenosynovitis	8 (50)	7 (43.8)	13 (81.3)
Synovitis	4 (25)	5 (31.3)	14 (87.5)
Erosions	1 (6.3)	1 (6.3)	10 (62.5)
BME (for MRI)	–	–	12 (75)

Percentage of agreement between the findings of MSUS and MRI is presented in Table 3.

**Table 3:** Percentage of agreement between the findings of MSUS and MRI

	Tenosynovitis	Synovitis	Erosion
MSUS (*n* = 16)	7	5	1
MRI (*n* = 16)	13	14	10
Percentage of agreement	62.5	43.8	43.8

### RAMRIS scores

The RAMRIS score for synovitis was 12.9 (range 8–19), for edema was 3.8 (range 1–8), and for erosions was 10.2 (range 4–16).

### Disease characteristics and synovial inflammation

There was significant correlation between disease duration, patients’ age, and MRI erosions (*p* = 0.001 and *p* = 0.01), respectively.

Also, significant correlation found between MSUS tenosynovitis and CRP (*p* = 0.04).

There were significant correlations between the CRP and the MRI tenosynovitis, MRI synovitis, and bone marrow edema (*p* = 0.002, *p* = 0.02, and *p* = 0.03), respectively.

RAMRIS synovitis score was significantly correlated with ESR (*p* = 0.3) and RAMRIS erosion score was significantly correlated with MRI findings of erosions and bone marrow edema (*p* = 0.004 and *p* = 0.04), respectively.

Disease subtype and antibody status did not have any significant relation to inflammation seen on MRI or MSUS or erosions.

## Discussion

Musculoskeletal findings in SSc have been the subject of continued controversy, partly because most reports describe mixed populations of PSS, mixed connective tissue disease (MCTD), and overlap syndromes of several connective tissue diseases [2].

Generalized arthralgia and morning stiffness are typical symptoms of systemic sclerosis and may be confused with early RA [3]. Erosive changes have been reported on X-rays in some SSc patients [5] and have been attributed to overlap with RA or MCTD [6].

In this study, we use MSUS and MRI to search for evidence of an inflammatory arthropathy in a group of SSc patients with arthralgia without clinical evidence of inflammatory arthritis and without an overlap with RA or patients with MCTD.

Although in our study, patients with positive RF and/or anti-CCP were excluded; there were 2/20 (10 %) patients who were RF positive and another 2/20 (10 %) patients who were anti-CCP positive, and those patients were excluded to allow a reasonable assumption that our patients did not include patients with clinically apparent RA overlap. However, Santiago et al. [14] found that the frequency of anti-CCP2 antibodies in SSc was 14.8 %. Varga and Denton [3] confirmed that RF positivity may be found in up to 30 % of patients with SSc.

In our study, anti-RNP was positive in 2/16 (12.5 %) patients; however, it did not have any significant relation to inflammation seen on MRI or MSUS or erosions. In agreement with these findings, Chitale et al. [4], who found anti-RNP antibody positive in 2/17 (14 %) patients and the antibody did not have any significant relation to inflammation seen on MRI or MSUS. Also, Varga and Denton [3] confirmed that about 20 % of patients with SSc have antibody directed against nuclear ribonucleoprotein (anti-RNP).

In our study, plain radiographic examination of both hands and wrists did not confirm the presence of erosive arthritis; this may be due to the fact that in SSc patients, the inflammation is usually low grade and is replaced by fibrosis at some stage [4]. However, Sari-Kouzel et al. [5] confirm the presence of erosions in hands and feet joints of SSc patients as seen on X-ray (with or without overlap syndrome), but synovitis has been little studied [4].

Chitale et al. [4] were the first to use MSUS to detect synovial inflammation and confirm its persistent nature in patients with SSc, and then, they compare their results with MRI.

In our study, we found the evidence of an inflammatory arthropathy in a group of SSc patients with arthralgia without clinical evidence of inflammatory arthritis using MSUS and MRI.

In agreement with these findings, Rodnan [15] reported that SSc patients had inflammatory changes in the synovium on biopsies. Schumacher [16] reported fibrin deposition and mild focal proliferation of synovial lining cells, with perivascular infiltration of lymphocytes and plasma cells in a proportion of SSc patients, further supporting evidence of synovial inflammation. Misra et al. [17] characterize the arthritis in a group of 34 SSc patients and identified synovitis in 88 %. Bourty et al. [18] reported mild inflammatory changes in the joints of SSc patients on MRI scan.

Low et al. [19] reported more inflammatory changes on MRI, in a significant proportion of their symptomatic SSc patients. However, their study cohort included patients with clinically swollen joints and positive serology for RF suggesting inclusion of patients with features of RA overlap.

In our study, at the baseline examination, tenosynovitis was present in 8 (50 %) out of 16 patients and synovitis was present in 4 (25 %) patients. On the second MSUS examination after 6 months, tenosynovitis was present in 7 (43.8 %) out of 16 patients and synovitis was present in 5 (31.3 %) patients that indicate the presence and persistence of inflammatory arthropathy.

In agreement with these findings, Chitale et al. [4] found that MSUS identified inflammation in a high proportion of patients: Tenosynovitis was more common and seen in 8 (47 %) of the 17 and 6 (46 %) of the 13 patients at baseline and second MSUS, respectively, than synovitis, which was identified in 1 (6 %) of the 17 and 3 (23 %) of the 13 patients at baseline and second MSUS, respectively. There was 70 % agreement for detection of synovial inflammation between baseline and second MSUS [expected agreement 50 %, (*p* = 0.06)], suggesting that inflammatory joint disease and tendinopathy were persistent in many patients.

In our study, of the 16 patients examined by MRI on the same week with the second MSUS examination, tenosynovitis was present in 13 (81.3 %) out of 16, synovitis was present in 14 (87.5 %), and bone marrow edema was present in 12 (75 %) patients. MRI proved to be much more sensitive in detecting synovial inflammation than MSUS in our study.

In agreement with these findings, Chitale et al. [4] found that tenosynovitis was present in 7 (88 %) of the 8 patients and bone marrow edema was seen in 63 % of patients. However, they found synovitis in 100 % of the patients, but this may be due to the fact that MRI was done for only 8/17 patients in their study who had signs of synovial inflammation on MSUS examination, but in our study, MRI was done for all patients with or without signs of synovial inflammation on MSUS examination.

In our study, MSUS confirms the erosive nature of this inflammatory arthropathy in one patient only; however, MRI confirms the erosive nature of this arthropathy in large proportion of the studied group. At the baseline examination, erosions were found in only 1 (6.3 %) patient. On the second MSUS examination after 6 months, erosions were also found in only 1 (6.3 %) patient. On MRI examination, erosions were found in 10 (62.5 %) patients. MRI proved to be much more sensitive in detecting erosions than MSUS in our study.

In agreement with these findings, Chitale et al. [4] found erosions in 6 (75 %) of the 8 patients on MRI examination; however, in their study, MSUS failed to identify any erosion at either baseline or on second MSUS, and so they proved that MRI was more sensitive in detecting erosions than MSUS in our study.

In our study, the percentage of agreement between MSUS and MRI for the detection of tenosynovitis, synovitis, and erosion was 62.5, 43.8, and 43.8, respectively. These results were comparable with a degree of agreement with Chitale et al. [4] who found that the degree of agreement between MSUS and MRI for the examined parameters (tenosynovitis, synovitis, and erosion) was 62, 38, and 25, respectively.

In our study, the RAMRIS score for synovitis was 12.9 (range 8–19), for edema was 3.8 (1–8), and for erosions was 10.2 (range 4–16). This mean RAMRIS scores in our study fall within the range of scores obtained from early [20, 21] and established [22] RA.

In agreement with these findings, Chitale et al. [4] found that the RAMRIS score for synovitis was 12.6 [interquartile range (IQR) 8.6–16.7], for edema was 3.4 (IQR 0.19–6.6), and for erosions was 9.75 (IQR 2.8–16.7). So they suggested that the extent of the synovitis, bone marrow edema, and erosions may not be dissimilar between RA and the selected studied group of SSc patients.

Our study has a number of limitations. This is a small study and includes only 16 patients with SSc with varied disease duration. Also, only symptomatic patients with arthralgia were included in the study. We recommend that further studies can be done involving larger number of patients and a control group consisting of SSc patients without arthralgia.

## Abbreviations

MRI: Magnetic resonance imaging
MSUS: Musculoskeletal ultrasonography
OMERACT: Outcome measures in rheumatology clinical trials
PDUS: Power Doppler ultrasonography
RAMRIS: A semiquantitative MRI scoring system used in RA
SSc: Systemic sclerosis
